# Losartan Attenuates Insulin Resistance and Regulates Browning Phenomenon of White Adipose Tissue in *ob/ob* Mice

**DOI:** 10.3390/cimb43030128

**Published:** 2021-10-28

**Authors:** Hsuan-Miao Liu, Cheng-Hui Wang, Zi-Yu Chang, Tse-Hung Huang, Tzung-Yan Lee

**Affiliations:** 1 Graduate Institute of Traditional Chinese Medicine, School of Chinese Medicine, College of Medicine, Chang Gung University, Taoyuan City 333, Taiwan; miaowhale@gmail.com; 2 Graduate Institute of Clinical Medical Sciences, College of Medicine, Chang Gung University, Taoyuan City 333, Taiwan; risingsun4518@gmail.com; 3 Institute of Traditional Medicine, School of Medicine, National Yang-Ming Chiao Tung University, Taipei City 112, Taiwan; changzhi887@gmail.com; 4 Department of Traditional Chinese Medicine, Chang Gung Memorial Hospital, Keelung City 204, Taiwan; huangtsehung@gmail.com

**Keywords:** losartan, insulin resistance, obesity, macrophage polarization, mitochondrial function, white adipose browning

## Abstract

Insulin resistance (IR) is a villain role to the pathology of fatty liver diseases implicated in adipose tissue dysfunction, which is characterized by lipid droplets (LDs) accumulation and hypoxia-inducible factor 1α (HIF1α) related macrophage infiltration. HIF1α is required for its lipogenic actions in adipocytes, while and it regulates M1 and M2 polarization features of macrophages. Losartan has been shown to be an insulin sensitizer in obese states, actions involving in HIF1α signaling. However, the exact mechanisms accounting for these effects have not been fully elucidated. Therefore, GTT, ITT, and HOMA-IR were identified losartan alleviated IR signaling in obese mice. This alleviation may through inhibits HIF1α by suppressing STAT3-NF-κB signaling, which, in turn, revealed HIF1α-dependent decreases the angiogenesis pathway in adipose tissue, including regulation of VEGF and TGFβR2 levels. In white adipose tissue, a set of lipogenesis-related genes, *Srebp1*, *Fas*, and *Scd-1* were markedly downregulated after losartan intervention, as well as reduced LDs size and LD-associated proteins, perilipin family proteins (PLINs) compared with obese mice. Losartan abolished macrophage infiltration with upregulation of M2 and inhibition of M1 macrophage markers in obese mice. Our data suggest that losartan attenuated obese-induced fatty liver, linked to alleviating inflammation in adipose tissues and a shift in M1/M2 macrophage balance. Furthermore, losartan might improve mitochondria biogenesis by upregulating SIRT1, PGC1α, UCP1, and mRNA of *Tfam*, *Cd137*, *Tmem26*, *Ucp1* expression in white adipose tissue compared with the obese group. Taken together, losartan may improve IR and adipose dysfunction by inhibiting lipotoxicity and HIF1α pathways.

## 1. Introduction

Adipose dysfunction may influence glucose homeostasis, including increased adipose energy storage in obesity results in increased free fatty acid (FFA) flux to other tissues, which promote insulin resistance (IR) and potential adverse effects, referred to by some as “lipotoxicity” [1]. Both lipotoxicity and inflammation play a major role in adipose tissue dysfunction in development of metabolic diseases, such as nonalcoholic fatty liver disease (NAFLD) and IR [2]. Development of IR represents the decreased ability of insulin to metabolize glucose and is characterized by dyslipidemia, hyperglycemia, a compensatory hyperinsulinemia, elevated blood pressure, and abdominal obesity [3]. Inflammatory mediators and IR are therefore directly involved in the regulation of macrophage polarization and adipose tissue function. In adipose tissue, impaired insulin action allows for increased lipolysis, which will contribute to re-esterification of lipids in other tissues (such as liver) and further exacerbates IR [4]. Lipid droplets (LDs)-associated proteins, such as perilipins (PLINs), and the cell death-inducing DNA fragmentation factor-alpha-like effector (CIDE) proteins (e.g., Cidea, Cidec), that have appeared as major regulators of the formation of LDs and lipid storage the formation of LDs in adipocytes and hepatocytes [5,6].

In adipose tissue, adipocytes hypertrophy induced the expression of hypoxia-inducible factor-1α (HIF-1α) is known to enhance obesity, while HIF-1α induces LDs accumulation and mediates cross-linking of collagens for adipose tissue fibrosis [7,8], that balance of which is critical to maintaining healthy energy homeostasis [9]. In addition, signaling of hypoxia are required for its pro-lipogenic actions in obese adipose tissue, while HIF-1α regulates features of both M1 and M2 polarization in macrophages. On the other hand, IR promotes de novo lipogenesis in adipocytes, aggravates macrophage infiltration and inflammatory response. Excessive free fatty acids result in adipocyte hypertrophy, leading to changes in the release of adipokines and local hypoxia in adipose tissue. These lead to release of inflammatory signaling factors and recruit of more immune cells into adipose tissue, which, in turn, interfere with intracellular insulin signaling [10,11].

Moreover, the white adipose tissue (WAT) displays capillary rarefaction and evidence of hypoxia, which correlates with macrophage infiltration and inflammatory cytokine expression in controlling adipose tissue inflammation, as well as overall metabolic function in obese patients and mice [12]. Macrophage infiltration and activation in the adipose tissue has provided a link between inflammation and adipose dysfunction, which causes in IR through the inhibition of post-receptor signal transduction [11] that recruitment of adipose tissue macrophages (ATMs) is associated with ectopic lipid accumulation and IR. Macrophages are classified into two types; first, macrophage M1 macrophages expression the surface marker CD11c, CCR7, and produce proinflammatory cytokines, such as interleukin (IL)-1, IL-6, and TNF-α [12,13]. Second, anti-inflammatory M2 macrophages expression the surface marker CD163, CD206, and CD301, as well as produce proinflammatory cytokines, such as IL-10 [13,14]. In humans, expression of the M1 macrophages in WAT correlates with body mass index (BMI) and upon obesity, thereby promoting adipose tissue inflammation [12], therefore, that obesity may induce the accumulation of adipose tissue inflammation and macrophages in human [12,15,16].

Mitochondrial dysfunction with incomplete or reduced mitochondrial fatty acid oxidation (FAO) is correlated with diminished insulin signaling in lipid-induced inflammation of mice model [17]. In addition, HIF1α is stabilized and promoted mitochondrial complex IV dysfunction (decreased activity and stability) in age-dependent obesity [18,19]. Adipose HIF-1α causes obesity and associated metabolic dysfunction by suppressing brown adipose tissue thermogenesis [15]. Recent evidence shows that ectopic mitochondrial uncoupling protein 1 (UCP1), UCP2, and peroxisome proliferator-activated receptor gamma coactivator 1 (PGC-1α) expression along with a gene expression profile of brown adipocytes (BA) induces WAT to obtain BA tissue (BAT) features [16,20]. Thus HIF-1α is required and adequate to prompt WAT inflammation and IR [21]. However, regarding systemic mechanisms of adipose HIF-1α and insulin pathway induced macrophage polarization and mitochondrial dysfunction is not clear.

Clinical evidence shows that losartan enhanced insulin sensitivity in nondiabetic hypertensive patients [22]. Our previous study showed that losartan prevents hepatic steatosis and macrophage polarization by inhibiting HIF-1α in NAFLD [23]. In addition, losartan could reduce the LDs accumulation induced by angiotensin II in podocytes [24] and improved insulin sensitivity [25,26], as well as induced thermogenic beige adipocytes in the inguinal WAT of obese mice [27]. Here, we used *ob/ob* mice to investigate the relationship between lipotoxic-related IR and HIF1α-related inflammation in obesity model. We found that losartan (i) improved glucose tolerance test (GTT), insulin tolerance test (ITT), and homeostasis model assessment of insulin resistance (HOMA-IR); (ii) ameliorated LDs accumulation in liver and epididymis WAT (EWAT); (iii) suppressed M1/M2 macrophage ratio and inflammation in *ob/ob* mice; (iv) promoted mitochondrial biogenesis and browning phenomenon in EWAT. These data demonstrate that losartan is a potential therapy for the control of obesity-induced abnormal insulin sensitivity by regulating lipotoxic and HIF1α-dependent macrophage polarization. Therefore, we find the losartan has a new therapy function, suggesting that maybe enhance the beneficial effects in obesity-associated diseases.

## 2. Results

### 2.1. Losartan Improves Glucose Tolerance and Insulin Sensitivity in ob/ob Mice

**Figure 1:**
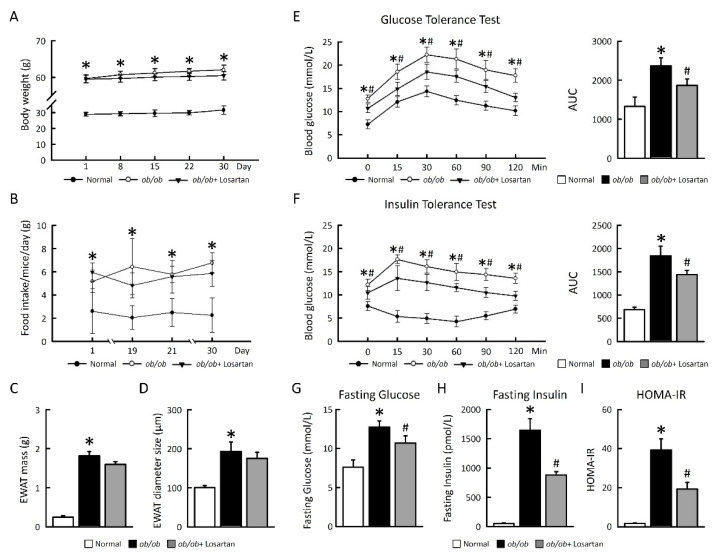
Losartan improved insulin sensitivity in EWAT. (**A**) 30-day Body weight change. (**B**) Food intake. (**C**) EWAT mass. (**D**) EWAT diameter size. (**E**) GTT (**F**) ITT. (**G**) Fasting glucose. (**H**) Fasting insulin. (**I**) HOMA-IR. Right graphs indicate quantification relative to AUC. For each animal group, *n* = 5. All values represent the mean ± SEM. Data were analyzed by Student’s *t* test. * *p* ≤ 0.05, normal vs. *ob/ob*; # *p* ≤ 0.05, *ob/ob* vs. *ob/ob*+ Losartan. HIF1α: hypoxia-inducible factor 1α; EWAT: epididymal white adipose tissue; GTT: glucose tolerance test; ITT: insulin tolerance test; HOMA-IR: homeostasis model assessment of insulin resistance.

To evaluate whether losartan ameliorates metabolic homeostasis, we studied the effects of losartan on glucose tolerance and insulin sensitivity in *ob*/*ob* mice. There was no significant change body weight, food intake, EWAT mass, and EWAT diameter size in *ob*/*ob* mice treated-losartan (Figure 1A–D). After 30 days losartan treatment, there was a significant improvement in glucose tolerance compared with *ob*/*ob* mice (Figure 1E). To assess whole-body insulin sensitivity, we performed insulin tolerance tests (ITTs), fasting glucose, fasting insulin, and HOMA-IR after treatment of losartan. There was a significant enhance in insulin sensitivity in *ob*/*ob* mice receiving losartan treatment compared with *ob*/*ob* mice (Figure 1F–I). These data demonstrate that losartan treatment of *ob*/*ob* mice results in a dramatic improvement in insulin sensitivity and whole-body glucose homeostasis.

### 2.2. Losartan Reduces Lipogenesis in EWAT of ob/ob Mice

**Figure 2:**
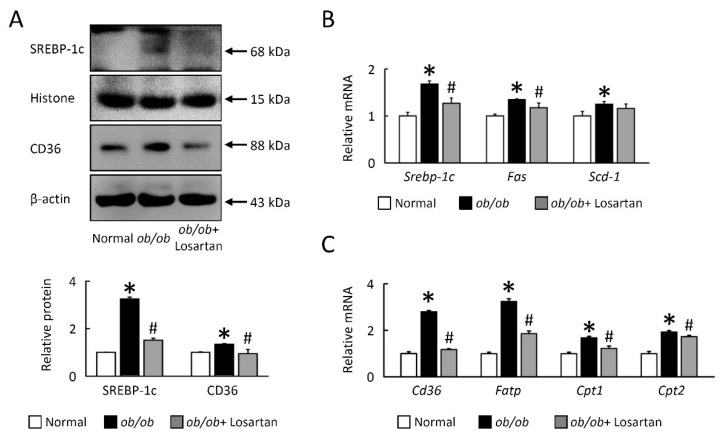
Losartan attenuates lipogenesis in EWAT. Quantification of (**A**) SREBP-1c, Histone, CD36, and β-actin protein levels by Western blot. Below graph indicate quantification relative to Histone/β-actin. Quantification of (**B**) *Srebp-1c*, *Fas*, *Scd-1*, (**C**) *Cd36*, *Fatp*, *Cpt1*, and *Cpt2* by qRT-PCR. qRT-PCR indicates quantification relative to *Gapdh*. For each animal group, *n* = 5. All values represent the mean ± SEM. Data were analyzed by Student’s *t* test. * *p* ≤ 0.05, normal vs. *ob/ob*; # *p* ≤ 0.05, *ob/ob* vs. *ob/ob*+ Losartan. *Srebp-1c*: sterol regulatory element-binding protein 1c; *Fas*: fatty acid synthase; *Scd-1*: stearoyl-CoA desaturase-1, *Fatp*: fatty acid transport protein; *Cd36*: cluster of differentiation 36; *Cpt1*: carnitine palmitoyltransferase 1.

In order to clarify that losartan did not significantly reduce weight but improved insulin resistance (IR), we next investigated cellular mechanisms for the effects of losartan on EWAT lipid accumulation. Proteins and genes expression of key molecules involved in lipogenesis (including sterol response element-binding protein (*Srebp*)-1c, fatty acid synthase (*Fas*) and stearoyl-CoA desaturase (*Scd-1*)), fatty acid uptake (cluster of differentiation 36 (*Cd36*), fatty acid transport protein (*Fatp*)), and oxidation (carnitine palmitoyl transferase 1 (*Cpt1*) and *Cpt2*) were decreased in the EWAT of *ob/ob* mice treated with losartan (Figure 2A–C). These data indicate that losartan is involved in the SREBP-1c-dependent pathway of obesity-mediated lipogenesis and that the potential effectors of lipogenesis are lipid droplets (LDs) sensitive.

### 2.3. Losartan Attenuates HIF1α Expression and LDs Formation in EWAT

**Figure 3:**
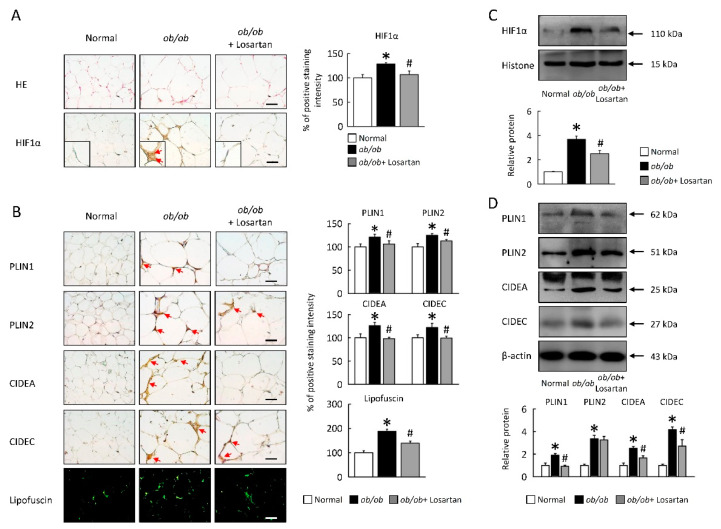
Losartan attenuated HIF1α expression and LDs formation in EWAT. (**A**) Representative HE and HIF1α staining. Red arrow highlights the positive staining. Scale bar: 100 μm. Tissue staining, as quantified by either stain intensity, is represented on the left-hand vertical axis in each graph. (**B**) Representative PLIN1, PLIN2, CIDEA, and CIDEC staining of EWAT. Green pseudocolor represents visualization of lipofuscin’s autofluorescence at 450–490 nm. Red arrow highlights the positive staining. Scale bar: 100 μm. Tissue staining, as quantified by either stain intensity, is represented on the left-hand vertical axis in each graph. Quantification of (**C**) HIF1α protein levels by Western blot. Below graph indicate quantification relative to Histone. Quantification of (**D**) PLIN1, PLIN2, CIDEA, and CIDEC protein levels by Western blot of EWAT. Below graphs indicate quantification relative to β-actin. For each animal group, *n* = 5. All values represent the mean ± SEM. Data were analyzed by Student’s *t* test. * *p* ≤ 0.05, normal vs. *ob/ob*; # *p* ≤ 0.05, *ob/ob* vs. *ob/ob*+ Losartan. HIF1α: hypoxia-inducible factor 1α; LDs: lipid droplets; EWAT: epididymal white adipose tissue; HE: hematoxylin and eosin stain; PLIN1: perilipin 1; CIDEA: cell death inducing DFFA like effector A.

To investigate the role of losartan on HIF1α regulates lipogenesis and LDs formation by studying (*ob/ob*) mice. HIF1α protein level was increased in *ob/ob* mice, losartan significantly reduced HIF1α protein level in EWAT of *ob/ob* mice (Figure 3A,B). Moreover, PLIN1, PLIN2, CIDEA, and CIDEC were also reduced in EWAT of *ob/ob* mice treated with losartan (Figure 3B,D). These data were further confirmed by histological analysis, highlighting a novel role for losartan in decreasing lipogenesis and LDs formation in the context of obesity.

### 2.4. Losartan Attenuates NF-κB/HIF1α/STAT3 and Angiogenesis Pathways in EWAT

**Figure 4:**
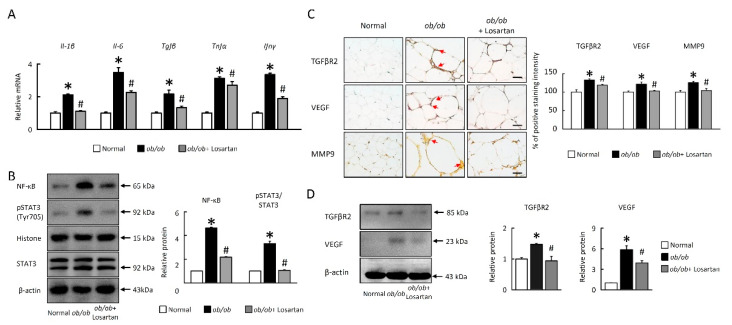
Losartan suppressed inflammatory response and angiogenesis in EWAT. (**A**) Quantification of *Il-1β*, *Il-6*, *Tgfβ*, *Tnfα*, *Ifnγ* by qRT-PCR. qRT-PCR indicates quantification relative to *Gapdh*. (**B**) Quantification of NF-κB, pSTAT3, STAT3, and β-actin protein levels by Western blot of EWAT. Below graphs indicate quantification relative to Histone (for NF-κB) and STAT3 (for pSTAT3). (**C**) Representative TGFRβ2, VEGF, and MMP9 staining of EWAT. Red arrow highlights the positive staining. Scale bar: 100 μm. Tissue staining, as quantified by either stain intensity, is represented on the left-hand vertical axis in each graph. (**D**) Quantification of TGFβR2 and VEGF protein levels by Western blot of EWAT. Below graphs indicate quantification relative to β-actin (for TGFRβ2 and VEGF). For each animal group, *n* = 5. All values represent the mean ± SEM. Data were analyzed by Student’s *t* test. * *p* ≤ 0.05, normal vs. *ob/ob*; # *p* ≤ 0.05, *ob/ob* vs. *ob/ob*+ Losartan. EWAT: epididymal white adipose tissue; *Il-6*: interleukin-6; *Tgfβ*: transforming growth factor beta; *Tnfα*, tumor necrosis factor α; *Ifnγ*: interferon gamma; TGFβ: transforming growth factor beta; VEGF: vascular endothelial growth factor; MMP-9 matrix metallopeptidase 9.

To examine further whether losartan in EWAT controls inflammation, we observed proinflammatory cytokine interleukin (*Il*)-*1β*, *Il-6*, transforming growth factor beta (*Tgfβ*), *Tnfα*, and interferon gamma (*Ifnγ*) mRNA expression were significantly increased in *ob*/*ob* mice (Figure 4A). In the results, the inflammatory genes expressions in *ob*/*ob*+ losartan group were significantly normalized compared to the *ob*/*ob* group. Losartan was also significantly suppressed nuclear factor κB (NF-κB), 5hosphor-STAT3 protein levels (Figure 4B) and proinflammatory cytokines expression (Figure 4B) in EWAT of *ob*/*ob* mice. Furthermore, we confirmed that losartan attenuated angiogenesis markers, transforming growth factor beta receptor 2 (TGFβR2), vascular endothelial growth factor (VEGF), and matrix metallopeptidase 9 (MMP-9) protein levels in EWAT of *ob*/*ob* mice (Figure 4C,D). These results suggest that IR due to lipotoxicity and HIF1α signaling pathways signaling may cause inflammation upon obesity, but treatment with losartan reversed this phenomenon.

### 2.5. Losartan Decreased M1 Macrophage Polarization in EWAT

**Figure 5:**
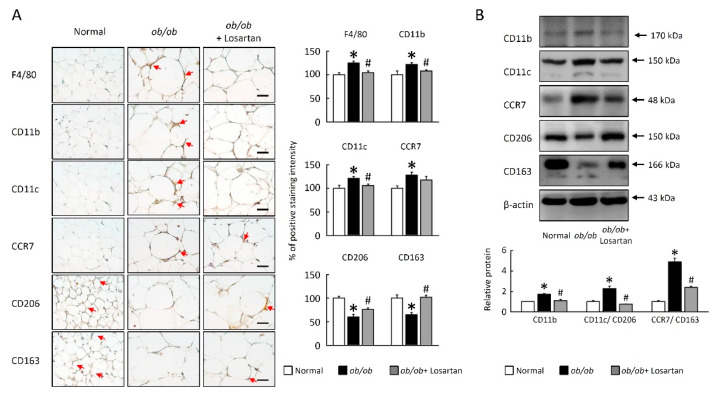
Losartan regulated macrophage polarization in EWAT. (**A**) Representative F4/80, CD11b, CD11c, CCR7, CD206, and CD163 staining of EWAT. Red arrow highlights the positive staining. Scale bar: 100 μm. Tissue staining, as quantified by either stain intensity, is represented on the left-hand vertical axis in each graph. (**B**) Quantification of CD11b, CD11c, CCR7, CD206, CD163, and β-actin protein levels by Western blot. Ratio of CD11c/CD206 and CCR7/CD163 in the EWAT. For each animal group, *n* = 5. All values represent the mean ± SEM. Data were analyzed by Student’s *t* test. * *p* ≤ 0.05; normal vs. *ob/ob*. # *p* ≤ 0.05; *ob/ob* vs. *ob/ob* + Losartan. EWAT: epididymal white adipose tissue.

The inflammatory cytokine of the adipocytes is possibly driven by increased expression of the macrophage infiltration and M1 macrophage activation. Next, we determined whether the increased macrophages in EWA of *ob/ob* mice were due to an increase in pro-inflammatory M1 and/or anti-inflammatory M2 macrophages. As shown in Figure 5, F4/80, CD11b, CD11c, and CCR7 levels were higher in *ob/ob* mice compared with normal mice (Figure 5A,B). Both macrophage infiltration (F4/80, CD11b) and M1 macrophage markers (CD11c, CCR7) were significantly lower in losartan treated mice (Figure 5A,B).

The expression of M2 macrophage surface markers CD206 and CD163 levels were lower in *ob/ob* mice compared with normal mice (Figure 5A,B). In contrast, both CD206 and CD163 were significantly higher in losartan-treated mice. These results showed that losartan attenuated the ratio of M1 to M2 in EWAT of *ob/ob*.

### 2.6. Losartan Improves Mitochondrial Dysfunction and Enhances Browning in EWAT

**Figure 6:**
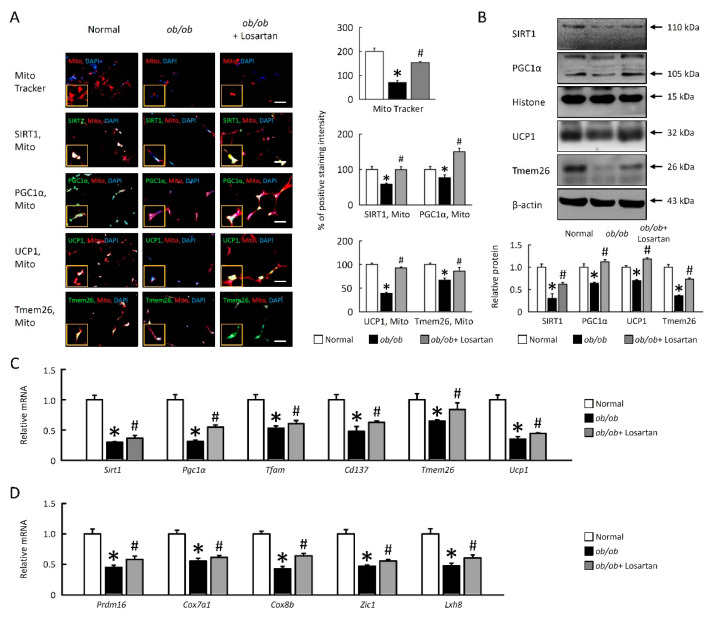
Losartan enhanced mitochondrial function and EWAT browning in *ob/ob* mice. (**A**) The staining for SIRT1, PGC1α, UCP1, and Tmem26 are green, while the staining for mitochondria (Mito Tracker) is red. Scale bar: 100 μm. Tissue staining, as quantified by either stain intensity, is represented on the left-hand vertical axis in each graph. (**B**) Quantification of SIRT1, PGC1α, UCP1, and Tmem26 protein levels by Western blot of EWAT. Below graphs indicate quantification relative to Histone (for SIRT1 and PGC1α) and β-actin (for UCP1 and Tmem26). (**C**) Quantification of *Sirt1*, *Pgc1α*, *Tfam*, *Cd137*, *Tmem26*, *Ucp1*, (**D**) *Prdm16*, *Cox7a1*, *Cox8b*, *Zic1*, and *Lxh8* by qRT-PCR. qRT-PCR indicates quantification relative to *Gapdh*. For each animal group, *n* = 5. All values represent the mean ± SEM. Data were analyzed by Student’s *t* test. * *p* ≤ 0.05, normal vs. *ob/ob*; # *p* ≤ 0.05, *ob/ob* vs. *ob/ob*+ Losartan. EWAT: epididymal white adipose tissue; SIRT1: sirtuin 1; PGC1α: peroxisome proliferator-activated receptor gamma coactivator 1-alpha; UCP1: uncoupling protein 1; *Tfam*: mitochondrial transcription factor A; *Prdm*: PR domain zinc finger protein 16; *Cox7a1*: cytochrome c oxidase; *Zic1*: Zinc finger of the cerebellum 1; qRT-PCR: Quantitative real time polymerase chain reaction.

Mitochondrial dysfunction has been involved in the pathogenesis of IR, we hypothesis boosting mitochondrial function may display a beneficial therapeutic tool to improve IR. To identify losartan may enhance mitochondrial function, we observed mitochondrial biogenesis markers sirtuin 1 (SIRT1), peroxisome proliferator-activated receptor gamma coactivator 1-alpha (PGC1α), and uncoupling protein 1 (UCP1) were enhanced in *ob/ob* mice-treated losartan (Figure 6A–C).

To further examine how losartan affects EWAT on a molecular level, EWAT samples were taken from normal mice, *ob/ob* mice and *ob/ob* mice-treated losartan. Consistent with this concept, SIRT1, PGC1α, UCP1, transmembrane protein 26 (Tmem26) protein, and mRNA expression were increased in EWAT of *ob/ob* mice treated with losartan (Figure 6A,B). Quantification of thermogenic genes *Sirt1*, *Pgc1α*, mitochondrial transcription factor A (*Tfam*), *Cd137*, *Tmem26*, *Ucp1*, PR domain containing 16 (*Prdm16*), cytochrome c oxidase polypeptide 7A1 (*Cox7a1*), Cox8b, Zinc finger protein 1 (*Zic1*), and *Lxh8* mRNA expression were also significantly increased in *ob/ob* mice treated with losartan (Figure 6C,D). Accordingly, these data demonstrated that losartan enhanced mitochondrial biogenesis and browning in EWAT of *ob/ob* mice.

### 2.7. Losartan Suppresses LDs Accumulation and Lipogenesis in Liver

**Figure 7:**
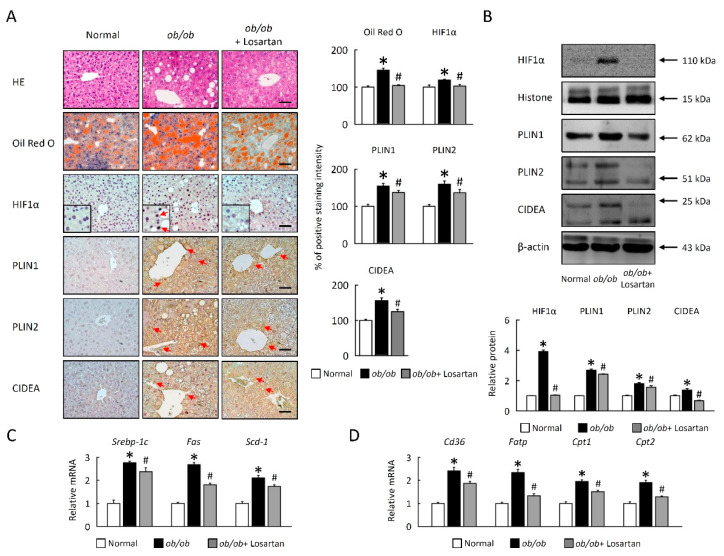
Losartan reduced LDs accumulation and lipogenesis in liver. (**A**) Representative HE, Oil Red O, HIF1α, PLIN1, PLIN2, and CIDEA staining of liver. Red arrow highlights the positive staining. Scale bar: 100 μm. Tissue staining, as quantified by either stain intensity, is represented on the left-hand vertical axis in each graph. (**B**) Quantification of HIF1α, PLIN1, PLIN2, and CIDEA protein levels in homogenates of liver. Below graphs indicate quantification relative to Histone (for HIF1α) and β-actin (for PLIN1, PLIN2, and CIDEA). (**C**) Quantification of *Srebp-1c*, *Fas* and *Scd1*, (**D**) *Cd36*, *Fatp*, *Cpt1*, and *Cpt2* by qRT-PCR. For each animal group, *n* = 5. All values represent the mean ± SEM. Data were analyzed by Student’s *t* test. * *p* ≤ 0.05, normal vs. *ob/ob*; # *p* ≤ 0.05, *ob/ob* vs. *ob/ob*+ Losartan. HIF1: hypoxia-inducible factor 1; LDs: lipid droplets; HE: hematoxylin and eosin stain; PLIN1, perilipin 1; CIDEA, cell death inducing DFFA like effector A; SREBP: sterol response element-binding protein; *Fas*: fatty acid synthase; *Scd*: stearoyl-CoA desaturase; *Cd36*: cluster of differentiation 36; *Fatp*: fatty acid transport protein; *Cpt1*: carnitine palmitoyltransferase 1; qRT-PCR: quantitative real time polymerase chain reaction.

Obesity-induced systemic insulin resistance, in general, is critically dependent on intracellular LDs accumulation and lipogenesis. To examine the possibility that losartan improved hepatosteatosis could involve the regulation of LDs accumulation and lipogenesis in liver, *ob/ob* mice were treated losartan. HIF1α, PLIN1, PLIN2, and CIDEA protein levels were reduced in liver of *ob/ob* mice treated with losartan (Figure 7A,B). These data were further confirmed by HE and Oil Red O staining, highlighting a novel role for losartan in reducing LDs accumulation in the context of obesity (Figure 7). We next investigated cellular mechanisms for the effects of losartan on hepatic lipid accumulation. Gene expression of lipogenesis (*Srebp-1c*, *Fas,* and *Scd1*), fatty acid uptake (*Cd36*, *Fatp*), and oxidation (*Cpt1* and *Cpt2*) were significantly decreased in liver of *ob/ob* mice treated with losartan (Figure 7C,D), suggesting losartan reduces LDs accumulation could be through suppression of de novo lipogenesis. Thus, we identified losartan is an important regulator of HIF1α, and lipid metabolism in liver of *ob/ob* mice.

### 2.8. Losartan Attenuates M1/M2 Macrophage Ratio in Liver

**Figure 8:**
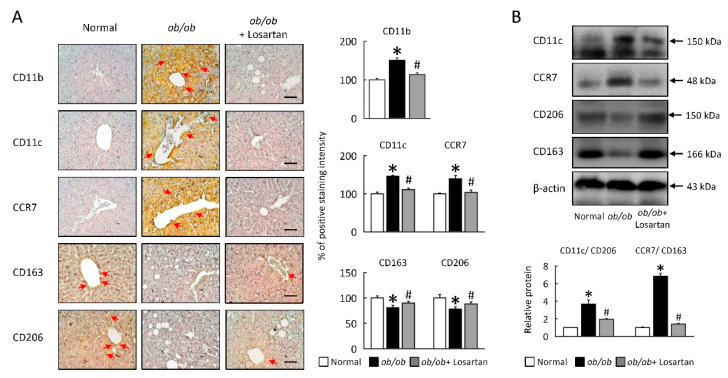
Losartan regulated macrophage polarization in liver. (**A**) Representative CD11b, CD11c, CCR7, CD163, and CD206 staining of liver. Red arrow highlights the positive staining. Scale bar: 100 μm. Tissue staining, as quantified by either stain intensity, is represented on the left-hand vertical axis in each graph. (**B**) Quantification of CD11c, CCR7, CD206, and CD163 protein levels by Western blot of liver. Below graphs indicate quantification relative to β-actin. Ratio of CD11c/CD206 and CCR7/CD163 in liver. For each animal group, *n* = 5. All values represent the mean ± SEM. Data were analyzed by Student’s *t* test. * *p* ≤ 0.05, normal vs. *ob/ob*; # *p* ≤ 0.05, *ob/ob* vs. *ob/ob* + Losartan.

To further verify whether obesity-induced system IR is mediated by activation of HIF1α-regulated macrophage polarization in liver, *ob/ob* mice were treated losartan. Losartan inhibited macrophage infiltration and M1 macrophage polarization in liver of *ob/ob* mice. In addition to improved liver macrophage infiltration and M1 macrophage polarization was also quantified based on levels of hepatic macrophage infiltration density in steatosis area, strong CD11b, CD11c, and CCR7 staining and protein levels were observed central tracks and steatosis area in the liver of *ob/ob* mice (Figure 8A,B). In contrast, *ob/ob* mice treated losartan showed a significant decrease in macrophage infiltration and M1 macrophage activation. Hepatic M2 macrophage, CD206 and CD163 were significantly increased in losartan treated *ob/ob* mice (Figure 8A,B). We also performed a Western blot analysis to determine the ratio of M1/M2 macrophage was affected, suggesting that suppression of M1/M2 macrophage ratio also contributed to the reduced obese effects of losartan.

## 3. Discussion

The variation of the endocrine functions and metabolic of adipose tissue is constantly associated with type 2 diabetes and IR. In the present study, we examined the therapeutic potential of losartan on IR and obesity which are mainly implicated in the deleterious metabolic cascade between hypoxia and mitochondria dysfunction. To our knowledge, there is no specific study so far of losartan on macrophage depolarization linked to mitochondria biogenesis in adipose tissue of *ob/ob* mice. Therefore, our overall aim was to specifically examine the role of losartan in WAT browning in the IR cascade of obese with specific implication in the adipose tissue. As such, losartan attenuated obese-stimulated fatty acid uptake activity in adipose tissue via decreased fatty acid transport protein and oxidation, which, in turn, reduced macrophage polarization and inflammatory response in adipose tissue from the obese mice.

In the current study, the used losartan dosage was well-tolerated and exceptionally safe, which was confirmed by no significant changes in body weight and serum levels of hepatic markers, AST and ALT, suggesting no hepatotoxicity exerted by losartan dosages in the current study upon obese feeding in mice. Obesity may disturb metabolic organs, such as liver and adipose tissues. In this regard, the alterations of several important adipokines or pro-inflammatory cytokines are closely linked to development of obesity-associated disorders and IR. In the present study, *ob/ob* mice significantly increased food intake, body weight gain and EWAT weight. In addition, the blood biochemical profiles including fasting glucose, fasting insulin and HOMA-IR were unfavorably altered, thus confirming the state of IR and obesity. The current study invented that losartan was able to recover these metabolic parameters with significant decreases in adipose tissue compared to the obese group, despite no significant differences in the food and daily energy intakes among treatment groups. Accordingly, a previous report found that in non-diabetic hypertensive patients, losartan reduced the insulin resistance index, HOMA-IR [22]. There is also consistent study support that angiotensin receptor blockers (ARBs) including losartan decrease the IR [28]. Nishimura et al. studied the effects of losartan (50–100 mg/day) versus a calcium channel blockers (CCBs) administered for 3 months in patients of impaired glucose tolerance. Losartan caused a significant reduction in HOMA-IR (23.9%) [29]. Consistently, the current study indicated that in vivo administration of losartan significantly decreased lipogenesis-stimulated fatty acid uptake in adipose tissue of the obese mice, implying such correlative effect of losartan in modulating fatty acid metabolism with reducing fasting blood glucose level through improved insulin sensitivity.

Obesity-induced chronic inflammation appears to negatively affect several insulin-sensitive tissues (e.g., adipose tissue, liver, and muscle) in individuals with IR and metabolic disorders. WAT also acts as an endocrine organ, releasing bioactive substances that have been coined adipokines, these include TNF-α, IL-6, IL-1β, leptin, and adiponectin [10], especially TNF-α on the functions of adipose tissue including induction of inhibition of insulin signaling, lipolysis, and alterations in the expression of adipocyte important genes through activation of NF-κB in adipose tissue of obese mice and linked to IR [30]. Adipose tissue lipid accumulation is accompanied not only by inflammatory response but also by extensive EWAT angiogenesis. Obese subjects exhibit adipose tissue hypoxia and induce HIF1α expression, which is a positive regulator of LDs producing, causes enhanced fatty acid uptake and lipid storage [12,13]. LD-associated protein, PLIN1 is a major regulatory protein for adipose metabolism, where, PLINs are favoring fat deposition via preventing lipolysis in basal conditions. PLIN deficient mice show a lean phenotype, resistant to diet-induced, or genetic obesity, and had peripheral IR [31]. Moreover, the common variations of the PLIN gene have been associated with diabetes, which is caused by weight gain, IR, obesity, and hypertension [32,33].

Indeed, the in vivo and in vitro administration of losartan in mice could reduce several pro-inflammatory cytokines and revealed that losartan in cellular models of insulin resistance [32,33]. In the present work, losartan administration reversed mRNA levels of pro-inflammatory cytokine relative, inflammatory state, and NF-κB activity in adipose tissue from the *ob/ob* mice. In adipose tissues and macrophages, macrophage infiltration and polarization are played a major role in the development of inflammation. Macrophage polarization is characterized as a metabolic phenotype switching between pro-inflammatory M1 (classical) and anti-inflammatory M2 (alternative) classes [34]. M1 macrophage polarization is mainly stimulated through adipose tissue inflammation, cause an elevated release of serum pro-inflammatory cytokines to promote IR in obesity [35]. These are in line with our current report finding that losartan treatment enhanced the M2 markers and reduced M1 markers expression in macrophages, which displays the beneficial effect of losartan in reducing macrophage infiltrations in obese mice. In the present study, losartan remarkably decreased HIF1α levels, PLIN1, and CIDEA in adipose tissue from the obese mice. These losartan’s effects could be attributed by reduced lipogenesis and fatty acid uptake, thus confirming attenuated macrophage polarization with reduced inflammatory response by losartan treatment in vivo. Therefore, we systematically indicated the correlative expression on the relative mRNA expression of these inflammatory cytokines in adipose tissue in response to losartan administration. The decreased inflammatory cytokines were associated with the reduced NF-κB/STAT3 phosphorylated activity. Moreover, the improvement of inflammatory response applied by losartan is metabolically linked to the direct modulation of inflammatory signaling in the crosstalk between adipose tissues and macrophages.

Reactive oxygen species (ROS) is a major risk of mitochondrial dysfunction and is also a consequence of inflammation cascade, with an implied role in the stabilization of HIF and induction of IR. There are several effector pathways elicited by mitochondrial dysfunction, including the AMPK pathway that can promote IR and reflects the metabolic state of cells [36], chronic low-grade inflammation and hypoxia promote the adipose tissue fibrosis and related metabolic dysfunctions process [37], which is implicated in insulin resistance initiation and progression, and the proinflammatory macrophages which is involved in obese development [38]. This study provides a potential therapeutic view of losartan initiated by mitochondrial dysfunction. Our previous findings established that losartan contributes to mitochondrial functions in NAFLD [23]. Here, our study demonstrated for the first time that losartan was able to decrease oxidative stress through regulation of SIRT1-Tfam-Tmem26-PGC-1α transcriptional axis with enhanced mitochondrial gene expression for thermogenesis in liver and adipose tissues. Our study illustrated that losartan treatment ameliorated obesity through significant improvement of mitochondrial functions via enhanced liver or adipose tissue antioxidant defense system of the *ob/ob* mice. The major regulators of mitochondrial biogenesis and oxidative metabolism include SIRT1, PGC-1α, and TFAM which have been involved in the development of obesity-mediated IR [39]. Moreover, the SIRT1-PGC1α mRNA expression level in adipose tissue was upregulated, suggesting losartan’s effect in improving mitochondrial biogenesis and function can be partly allied to improved glucose metabolism and fatty acid. AMPK is in response to ATP/AMP ratio in the cytoplasm, which is a highly conserved master transcriptional regulator of glucose and lipid metabolism [40]. AMPK promotes mitochondrial biogenesis in several peripheral tissues through direct phosphorylation of PGC1α in couple leading to increased expression of other transcription regulators, including NRF-1 and TFAM. Given this integrate association to mitochondrial functions biogenesis and functions, it seems plausible that losartan may perhaps apply some profound effects in vivo on the AMPK/SIRT1 pathways. Several reports indicated that the browning process occurs dependent on SIRT1/PGC1α signal in anatomically homogeneous adipose depots and a heterogeneous fashion within morphologically, that pre-adipocyte differentiation into brown (or “beige/brite”) adipocytes by activating of SIRT1/PGC1α and PPARγ deacetylation [41]. This is in accord with our results showing that mitochondria are significantly impaired in *ob/ob* mice [42] and that mitochondrial biogenesis (SIRT1, PGC1α) and producing browning in *ob/ob* mice treatment with losartan. Our present results are consistent with prior findings showing losartan supplementation in vivo selectively restored and increased SIRT1 expression by losartan leads to enhanced PGC-1α expression, and AMPK requires PGC-1α activity to modulate the expression of several key players in glucose metabolism and mitochondrial functions. In our experimental conditions, the markers for the perpetuation phase of EWAT browning, UCP1, *Cd137*, *Cox7a1*, and *Tmem26* were also increased in the presence of losartan. In parallel, losartan remarkably enhanced SIRT1 levels, leading to the deacetylation of PGC-1α transcriptional activities with decreased M1/M2 macrophage ratio. Mechanistically, the current study evaluated losartan supplementation correlative mechanism on the signaling among lipogenesis, adipose tissue browning, and mitochondria biogenesis in the adipose tissue of obese mice. These data support the hypothesis that losartan improved above signaling pathways, which are implicated in meet the energetic requirements of the tissues and improving mitochondrial respiration in circumstances of energy stress.

The current study evidenced the multifunctional roles of losartan against obesity-mediated metabolic dysfunction in concert with its beneficial effect in decreased lipotoxicity. This may, therefore, be a promising therapeutic strategy to impairment of metabolism and mitochondrial homeostasis is emphasized. Collectively, these results have shown that losartan ameliorative properties on the additional mechanistic against the development of IR and obesity.

## 4. Materials and Methods

### 4.1. Animals

The 20-week-old leptin-deficient (*ob/ob*) mice and C57BL/6J (normal) mice were obtained from the National Laboratory Animal Center (Taiwan) and maintained under a 12-h light/12-h dark cycle at 22 ± 2 °C for this study. Animals had free access to water and standard mouse chow. All experiments were performed in adherence with the “National Institutes of Health Guidelines on the Use of Laboratory Animals” and were approved by the Chang Gung University Institutional Animal Care and Use Committee. Fifteen mice were randomized into three experimental groups (*n* = 5/group): (1) normal mice; (2) *ob/ob* mice; (3) *ob/ob* mice + losartan (100 mg/L dissolved in water for 30 days), that, afterwards, were sacrificed by CO_2_.

### 4.2. Glucose Tolerance Test (GTT)

At 23 weeks of age, an intraperitoneal GTT was used to assess the response of mice to a glucose challenge. After a 6-h fast, mice were given an intraperitoneal injection of glucose at 1 g/kg body weight. Blood glucose levels were measured via tail clip immediately prior to the glucose bolus and then at 0, 15, 30, 60, 90 and 120 min after injection. Blood glucose levels were measured with a glucose analyzer (OneTouch^®^ Ultra, Lifescan, Johnson & Johnson, Milpitas, CA, USA).

### 4.3. Insulin Tolerance Test (ITT)

At 24 weeks of age, mice underwent an ITT. Mice were fasted for 6 h and insulin 0.75 U/kg body weight (Humilin; Lilly, Indianapolis, IN, USA) was administered by intraperitoneal injection. Blood glucose levels were monitored immediately prior to insulin injection and then at 0, 15, 30, 60, 90 and 120 min thereafter.

### 4.4. HOMA-IR

The homeostatic model assessment of insulin resistance (HOMA-IR) is calculated by fasting insulin and fasting glucose levels. Scores were calculated with the following equation: (blood glucose (mg/dL) × (serum insulin (uU/mL))/405) [43].

### 4.5. Histology, Immunohistochemistry, and Immunofluorescence

Adipose and liver tissue were fixed overnight in 4% formalin at room temperature, embedded in paraffin, and cut into 5-μm-thick sections. For histology, sections were attained with hematoxylin (2 g/L) for 15 min and with eosin (0.1%; in 0.0003% acetic acid) for 10 min. For immunohistochemistry, and immunofluorescence, sections were stained with anti-HIF1α (ab179483, abcam, Cambridge, UK), PLIN1 (Ab3526, abcam, Cambridge, UK), PLIN2 (Novus Biologicals, Centennial, CO, USA), CIDEA (Novus Biologicals, Centennial, CO, USA), CIDEC (Ab198204, abcam, Cambridge, UK), SIRT1 (ab110304, abcam, Cambridge, UK), PGC1α (ab54481, abcam, Cambridge, UK), UCP1 (ab10983, abcam, Cambridge, UK), Tmem26 (PA5-23477, Thermo Fisher Scientific, Waltham, MA, USA), HIF1α (ab179483, abcam, Cambridge, UK), TGFβR2 (SC-17791, Santa Cruz Biotechnology, Dallas, TX, USA), VEGF (ab69479, abcam, Cambridge, UK), MMP9 (AB19016, Millipore, Burlington, MA, USA), F4/80 (Ab6640, abcam, Cambridge, UK), CD11b (ab133357, abcam, Cambridge, UK), CD11c (ab52632, ab11029, abcam, Cambridge, UK), CCR7 (ab32527, abcam, Cambridge, UK), CD163 (ab182422, abcam, Cambridge, UK), CD206 (ab64693, abcam, Cambridge, UK). Secondary antibody conjugated with HRP-conjugated anti-rabbit (G-21234, Millipore, Burlington, MA, USA), anti-mouse (G-21040, Millipore, Burlington, MA, USA), anti-rat (31470, Genetex, Irvine, CA, USA) or anti-goat (31402, abcam, CA, UK) secondary antibody, followed by incubation in DAB peroxidase solution (Millipore, Burlington, MA, USA), and subsequent counterstaining with hematoxylin (Sigma, St. Louis, MO, USA). Images were obtained using Olympus IX71 microscope. Sections were incubated with the secondary antibody Alexa Fluor 488, Alexa Fluor Plus 647, Alexa Fluor 633, anti-mouse or anti-rabbit (A-11029, A11034, A32795, Thermo Fisher Scientific, Waltham, MA, USA) for immunofluorescence and DAPI (62248, Thermo Fisher Scientific, Waltham, MA, USA) for nuclear staining. We measured adipocyte diameter size in EWAT used Olympus cellSens. Positive staining for immunohistochemistry and immunofluorescence were quantified using ImageJ software (1.45, NIH).

### 4.6. Oil Red O (ORO) Staining

The LD were visualized by ORO staining (O0625; Merck KGaA, Darmstadt, Germany). A stock solution was prepared by dissolving 0.1 g in 20 mL 2-propanol (0.5% *w*/*v*). The working solution was obtained by diluting the stock solution 2:3 with distilled water, yielding a concentration of 0.2% oil red O in 40% 2-propanol. Fresh liver tissues were embedded carefully in optimal cutting temperature compound (OCT) in a plastic mold, followed by freezing at −80 °C. Liver tissue sections were stained with ORO working solution for 15 min, that was later rinsed with 50% isopropanol and counterstained with hematoxylin for the nucleus. Positive staining for ORO was quantified using ImageJ software (1.45, NIH).

### 4.7. Auto-Fluorescence Detection of Lipofuscin

Tissue sections were mounted into 40% glycerol/TBS mounting medium. Lipofuscin auto-fluorescence was then manifested by excitation at 450–490 nm, using a long-pass filter at 515 nm [44]. We used the Olympus microscope equipped with the equipped with the DP74 camera.

### 4.8. RNA Isolation and Quantitative Real-Time PCR

Total RNA was purified from tissues using TRIzol Reagent (Life Technologies, Carlsbad, USA) and Rneasy Kit (QIAGEN) from EWAT and liver. RNA was reverse transcribed to cDNA using a cDNA Synthesis Kit (Thermo Fisher Scientific, Waltham, MA, USA). Semiquantitative real-time PCR analysis was performed using Fast SYBR Green (ROCHE, Basel, Switzerland). Relative expression levels were determined by normalizing each Ct value to either gene expression for mice samples using the ΔΔCt method. The primer sequences are shown in Table 1.

### 4.9. Protein Isolation and Western Blot Analysis

Tissues were homogenized in lysis buffer (100 mM Tris-HCl, pH 7.6, 2 mM EDTA, 2 mM EGTA, 150 mM NaCl, 1% Triton X-100) containing proteinase inhibitors and phosphatase inhibitors (Thermo Fisher Scientific, Waltham, MA, USA). The soluble proteins were quantified with the Bio-Rad Rapid Coomassie kit (Bio-Rad, Hercules, CA, USA). Total protein (60–80 μg) was run on a 10% SDS-polyacrylamide gel and transferred onto 0.45-μm PVDF blotting membrane (GE Healthcare), and detected with a specific antibody against PLIN1 (ab3526, abcam, Cambridge, UK), PLIN2 (NB110-40877, Novus Biologicals, Centennial, CO, USA), CIDEA (NBP1-76950, Novus Biologicals, Centennial, CO, USA), CIDEC (ab198204, abcam, Cambridge, UK), SREBP-1c (SC-366, Santa Cruz Biotechnology, Dallas, TX, USA), CD36 (SC-70644, Santa Cruz Biotechnology, Dallas, TX, USA), SIRT1 (ab110304, abcam, Cambridge, UK), PGC1α (ab54481, abcam, Cambridge, UK), UCP1 (ab10983, abcam, Cambridge, UK), Tmem26 (PA5-23477, Thermo Fisher Scientific, Waltham, MA, USA), HIF1α (ab179483, abcam, Cambridge, UK), TGFβR2 (SC-17791, Santa Cruz Biotechnology, Dallas, TX, USA), VEGF (ab69479, abcam, Cambridge, UK), NF-κB (SC-8008, Santa Cruz Biotechnology, Dallas, TX, USA), p-STAT3 (05-485, Millipore, Burlington, MA, USA), STAT3 (06-596, Millipore, Burlington, MA, USA), CD11b (ab133357, abcam, Cambridge, UK), CD11c (ab52632, ab11029, abcam, Cambridge, UK), CCR7 (ab32527, abcam, Cambridge, UK), CD163 (ab182422, abcam, Cambridge, UK), CD206 (ab64693, abcam, Cambridge, UK), β-actin (MAB1501, Millipore, Burlington, MA, USA), Histone (SC-56616, Santa Cruz Biotechnology, Dallas, TX, USA). Membranes were blocked for 1 h at room temperature in 5% BSA (ROCHE, Basel, Switzerland) in TBS containing 0.1% Tween-20 (TBST), followed by overnight incubation at 4 °C with the primary antibody. Then, the membranes were washed with TBST and further incubated at room temperature for 1 h with an HRP-conjugated anti-rabbit (G-21234, Millipore, Burlington, MA, USA), anti-mouse (G-21040, Millipore, Burlington, MA, USA), anti-rat (31470, Genetex, Irvine, CA, USA) or anti-goat (31402, abcam, Cambridge, UK) secondary antibody. The protein expression was detected using an enhanced chemiluminescence kit (Millipore, Burlington, MA, USA), and quantified using ImageQuant 5.2 software. Each experiment was repeated with a minimum of 3 independently prepared protein samples.

### 4.10. Statistical Analysis

Data were presented as the means ± standard error of the mean (SEM). Statistical significance was calculated with either a 2-tailed Student’s *t* test. A *p* value of less than 0.05 was considered significant.

## Figures and Tables

**Table 1:** The primers sequence for qRT-PCR.

Gene	Forward	Reverse
*Srebp-1c*	5′ actgtcttggttgttgatgagctggagcat 3′	5′ atcggcgcggaagctgtcggggtagcgtc 3′
*Fas*	5′ tgtcattggcctcctcaaaaagggcgtcca 3′	5′ tcaccactgtgggctctgcagagaagcgag 3′
*Scd-1*	5′ ccggagaccccttagatcga 3′	5′ tagcctgtaaaagatttctgcaaacc 3′
*Fatp*	5′ gcttcaacagccgtatcctc 3’	5′ tcttcttgttggtggcactg 3’
*Cd36*	5′ gcaaaacgactgcaggtcaac 3′	5′ tggtcccagtctcatttagcca 3′
*Cpt1*	5′ ggacagagactgtgcgttcct 3′	5′ gcgatatccaacagtgcttga 3′
*Cpt2*	5′ caaggccctggctgatgatgtg 3′	5′ agtctctgtccgcccctctcg 3′
*Il-1β*	5′ aacctgctggtgtgtgacgttc 3′	5′ cagcacgaggcttttttgttgt 3′
*Il-6*	5′-gtactccagaagaccagagg-3′	5′-tgctggtgacaaccacggcc-3′
*Tgf* *β*	5′ tatagcaacaattcctggcg 3′	5′ tgctgtcacaggagcagtg 3′
*Tnf* *α*	5′ ttgacctcagcgctgagttg 3′	5′ cctgtagcccacgtcgtagc 3′
*Ifnγ*	5′ cctcaaacttggcaatactc 3′	5′ agcaacaacataagcgtcat 3′
*Sirt1*	5’ gcaacagcatcttgcctgat 3’	5′ gtgctactggtctcactt 3′
*Pgc1α*	5′ gactcagtgtcaccaccgaaa-3′	5′ tgaacgagagcgcatcctt 3′
*Tfam*	5′ ggaatgtggagcgtgctaaaa 3′	5′-tgctggaaaaacacttcggaata 3′
*Ucp1*	5′ cctgcctctctcggaaacaa 3′	5′-tgtaggctgcccaatgaaca 3′
*Tmem26*	5′ accctgtcatcccacagag 3′	5′ tgtttggtggagtcctaaggtc 3′
*Cd137*	5′ cgtgcagaactcctgtgataac 3′	5′ gtccacctatgctggagaagg 3′
*Prdm16*	5′ cagcacggtgaagccattc 3′	5′ gcgtgcatccgcttgtg 3′
*Cox7a1*	5′ cagcgtcatggtcagtctgt 3′	5′ agaaaaccgtgtggcagaga 3′
*Cox8b*	5′ gaaccatgaagccaacgact 3′	5′ gcgaagttcacagtggttcc 3′
*Zic1*	5′ ctgttgtgggagacacgatg 3′	5′ cctcttctcagggctcacag 3′
*Lxh8*	5′ acacgagctgctacattaagga 3′	5′ cccagtcagtcgagtggatg 3′
*Gadph*	5′ tcaccaccatggagaaggc 3′	5′ gctaagcagttggtggtgca 3′

*Srebp-1c*: sterol regulatory element-binding protein 1c; *Fas*: fatty acid synthase; *Scd-1*: stearoyl-CoA desaturase-1, *Fatp*: fatty acid transport protein; *Cd36*: cluster of differentiation 36; *Cpt1*: carnitine palmitoyltransferase 1; *Il-6*: interleukin-6; *Tgfβ*: transforming growth factor beta; *Tnfα*: tumor necrosis factor α; *Ifnγ*: interferon gamma; *Sirt1*: NAD-dependent deacetylase sirtuin-1; *Pgc1α*: peroxisome proliferator-activated receptor gamma coactivator 1-alpha; *Tfam*: mitochondrial transcription factor A; *Ucp1*: uncoupling protein 1; *Tmem26*: transmembrane protein 26; *Prdm16*: PR domain containing 16; *Cox7a1*: cytochrome c oxidase subunit 7A1; *Cox8b*: cytochrome c oxidase subunit 8B; *Zic1*: Zinc finger of the cerebellum 1; *Gadph*: glyceraldehyde-3-phosphate dehydrogenase.

## Data Availability

The data generated for this study are available from the corresponding author on reasonable request.
